# Desmoplastic melanoma: C>Ts and NF‐*κ*B

**DOI:** 10.1111/pcmr.12451

**Published:** 2016-01-20

**Authors:** Roy Rabbie, David J. Adams

The extremely high mutational load suggests that immunotherapy may be particularly effective in this disease.

The past 5 years have seen a paradigm shift in the survival of patients with advanced melanoma, owed in large part to an improved understanding of the genomic complexity of the disease and the development of novel therapies. Large international efforts to systematically sequence tumours have uncovered molecularly discrete subtypes of melanoma, with mutational characteristics found to segregate with tumour histology and presentation.

Desmoplastic melanoma (DM) is a rare melanoma subtype characterised by spindled malignant melanocytes and abundant desmoplastic stroma. These melanomas are usually amelanocytic with an appearance resembling scar tissue that can often lead to delayed or occasionally missed diagnoses (McCarthy et al., 2004). Current evidence suggests that genomic changes in DM are very different to that of conventional melanoma, although the only well‐established defining features are a markedly reduced frequency of activating *BRAF* mutations and loss‐of‐function mutations in *NF1* (Wiesner et al., 2015).

Importantly, a new study lead by researchers from the University of California, San Francisco (UCSF), and recently published in *Nature Genetics*, offers the first comprehensive analysis of the molecular landscape of DM, with implications for diagnosis and treatment. In this study, a total of 62 specimens were collected from three international centres including UCSF, Memorial Sloan Kettering in New York and the Melanoma Institute Australia, in Sydney. The investigators, led by Boris Bastian, performed next‐generation low‐coverage genome and high‐coverage exome sequencing of 20 fresh frozen tumours and matched normal DNA in a discovery cohort, followed by targeted sequencing of 293 candidate genes on a validation cohort of 42 formalin‐fixed primary samples. To identify potential tumour suppressor gene candidates, the investigators looked at genes with a disproportionately high frequency of loss‐of‐function mutations (truncating and potentially deleterious missense variants). In order to identify additional novel pathogenic mutations, the clustering of mutations at ‘hotspots’ was investigated.

The first striking discovery made by Shain et al. was the extraordinarily high number of mutations with a median of 62 mutations per megabase being reported, substantially higher than other cutaneous melanomas (about 15/Mbp), which firmly ranks DM among the most highly mutated cancers sequenced to date. The high abundance of cytosine‐to‐thymidine (C>T) transitions, particularly at dipyrimidines, implicates UV radiation as the dominant mutagen in DM. This should not come as a surprise in a cutaneous neoplasm associated with chronic UV exposure; however, it is of interest within this particular phenotypic subtype with a predominantly intradermal presentation and, as suggested by the authors, strongly implicates a superficially located primary cell of origin. The exceptionally high mutational burden also proffers the question of whether inherited defects in DNA repair or pigmentation may contribute to susceptibility, a question that could be explored using the germline genome sequences of this cohort.

The high mutational burden observed in DM is also of significance for patient management. The strong therapeutic effect of immune checkpoint blockade in some patients with melanoma has been linked to expression of neoantigens; mutant peptides presented by MHC Class 1. A higher overall mutational burden would be expected to lead to the expression of more neoantigens, with mutation number being associated with improved efficacy of immunotherapy. In keeping with this, a recent retrospective analysis of over 1000 patients receiving immunotherapy identified 23 patients with DM who received anti‐PD1/anti‐PDL1 antibodies (Eroglu et al., 2015). The investigators showed an exceptional RECIST response rate of 70% in this cohort, a frequency over double that observed in an unselected population with advanced melanoma.

The second key finding by Shain et al. was that the most common mutational hotspot in DM occurs in a promoter region that regulates the expression of the nuclear factor kappa‐light‐chain enhancer of activated B cells (NF*κ*B) inhibitor epsilon gene, *NFKBIE*. This locus was also amplified in 14.5% of samples. Intriguingly validation of *NFKBIE* promoter mutations revealed that both alleles of the gene were altered in some cases. The protein (I*κ*B*ε*) encoded by *NKFBIE* inhibits the activity of proinflammatory NF*κ*B transcription factors by sequestering them in the cytoplasm (Oeckinghaus and Ghosh, 2009). Using the Illumina Human Bodymap RNA sequencing data, the authors neatly demonstrated that the mutational hotspot was in the promoter region of the short isoform of *NFKBIE*, which they showed to be ubiquitously expressed in melanoma using data from The Cancer Genome Atlas (TCGA). Mutation of this hotspot region was also demonstrated in two melanoma cell lines (M257 and M375), which were both shown to express the same short isoform of *NFKBIE*, and showed a lack of NF*κ*B translocation to the nucleus. NF*κ*B mediates crosstalk between inflammatory signals and cancer at multiple levels and there is emerging in vivo evidence that inhibiting NF*κ*B signalling can elicit an antitumour immune response and can even enhance the effects of immune checkpoint inhibitors (Hayakawa et al., 2014). Thus, it follows that a mutated/amplified *NFKBIE* promoter leading to attenuated NF*κ*B signalling may participate in the recognition of DM cells by the immune system, although the exact functional role of *NFKBIE* in DM is yet to be explored.

In addition to *NFKBIE* promoter alterations, highly focal amplifications of *MAP3K1* were identified in three tumours, and two novel melanoma‐related genes were found: *CBL* and *FBXW7*. Other mutated genes included *NF1*,* CDKN2A, ERBB2*,* MAP3K1*,* EGFR*,* PTPN11*,* MET*,* RAC1*,* SOS2*,* NRAS* and *PIK3CA*. Mutations in *TP53* and amplification of *TERT* were also observed. Importantly, *TERT* promoter mutations or amplifications occurred in 90% of the DMs analysed in this study. Amplification and consequent changes in expression levels of EGFR, MET, CDK4, CCND1, YAP1 and MDM2 were shown. The study of these somatic alterations not only serves to provide a better understanding of the pathogenesis of DM, but also has implications for the use of molecular pathway inhibitors in the clinic.

This report represents a substantial leap forwards in deciphering the molecular mechanisms underlying DM and provides unequivocal evidence that DM is genomically distinct from common melanoma. The extremely high mutational load suggests that immunotherapy may be particularly effective in this disease, potentially paving the way towards a rational preselection strategy for the use of immune checkpoint blockade in patients with this disease. The finding of focal amplifications/promoter mutations of *NFKBIE* in DM is also of particular interest, providing a window to a distinct mechanism driving this subtype of melanoma.

This paper highlights the benefits of large‐scale genomic analyses in rare tumours types by showing that genomic alterations that might not have otherwise been detected with small studies can be found by collaborators leveraging sample collections between centres.
